# Facilitators and Barriers to Implementing Peer‐Led Neurocognitive Screening for Older Adults Living With HIV at a Community Hospital in Thailand: A Multiple‐Methods Study

**DOI:** 10.1002/jia2.70135

**Published:** 2026-06-11

**Authors:** Linda Aurpibul, Rattika Thammalangka, Pramual Threeyakul, Arunrat Tangmunkongvorakul, Nittaya Phanuphak, Morgan M. Philbin, Claude A. Mellins

**Affiliations:** ^1^ Research Institute for Health Sciences Chiang Mai University Chiang Mai Thailand; ^2^ Fang Hospital Chiang Mai Thailand; ^3^ Institute of HIV Research and Innovation Bangkok Thailand; ^4^ Department of Medicine University of California San Francisco San Francisco California USA; ^5^ Department of Psychiatry New York State Psychiatric Institute and Columbia University New York New York USA

**Keywords:** barriers, facilitators, HIV, implementation, neurocognitive, older adults, screening

## Abstract

**Introduction:**

Neurocognitive impairment (NCI) is increasingly prevalent among older adults living with HIV (OALHIV). HIV clinics in low‐ and middle‐income countries often do not routinely screen for NCI. Task‐shifting screening to peer educators might be an alternative approach. We explored facilitators and barriers to implementing a peer‐led NCI screening tool in an HIV clinic in Thailand.

**Methods:**

The study took place at a community hospital HIV clinic in suburban Chiang Mai. Peer educators were trained to screen OALHIV ≥50 years for NCI using the International HIV‐Dementia Scale for global cognition, the Trail‐Making Test for psychomotor speed and the General Practitioner Assessment of Cognition. We interviewed OALHIV who participated in the peer‐led NCI screening during and after a 12‐week implementation phase, peer educators and healthcare professionals to identify individual and intervention characteristics categorized according to the Consolidated Framework for Implementation Research. Transcribed and imported digital materials were used for thematic analysis in Dedoose (version 9.2.12).

**Results:**

From March to June 2023, 162 eligible OALHIV were recruited. Among the 144 (89%) screened, the median age was 58 (IQR 54−62) years, and 93 (65%) were female. On at least one measure, 40 (28%) matched the study‐defined referral criteria for NCI, of whom 20 (50%) were diagnosed with mild NCI, representing 14% of all those screened. We conducted 39 interviews with OALHIV (*n* = 26) and clinic‐based staff (*n* = 12; five peer educators, eight healthcare professionals). The perceived benefits of neurocognitive screening, the empowerment of peer educators, the reliance on established links between peers and clinic clients, and OALHIV acceptance of screening were facilitators of the intervention. The main reported barriers were a lack of perceived necessity among OALHIV, clinic staff concerns about screening quality and comprehensiveness, and a lack of supportive national policies to integrate screening into routine HIV care for OALHIV.

**Conclusions:**

We demonstrated that a peer‐led NCI screening intervention was feasible and acceptable in our setting. Raising awareness of the benefits of NCI screening among OALHIV and improved training and coordination in the healthcare setting would facilitate more effective implementation.

## 1 Introduction

The life expectancy of people living with HIV has improved with effective treatment and care [1]. However, they remain at a higher risk of neurocognitive disorders due to (1) central nervous system invasion of HIV, (2) neurotoxic viral protein effects on the blood−brain barrier and (3) persistent neuroinflammation [2], including neurocognitive impairment (NCI) [3]. Immunosenescence and mitochondria dysfunction accelerate ageing and neurocognitive deterioration [4], as does the chronic use of antiretroviral therapy (ART) [5]. Clinical symptoms of NCI involve deficits in concentration, attention and memory, all of which can affect ART adherence [6]. According to a study across eight countries in the Asia‐Pacific region, the prevalence of moderate to severe NCI among people living with HIV was approximately 12% [7]; and is expected to increase as people continue to age.

In 2023, approximately 580,000 adults in Thailand were living with HIV [8]. Per the National AIDS Program, 41% were over 40 (57% male), and 21% were over 50 years old (55% male) [9]. The prevalence of NCI among middle‐aged Thai people with HIV receiving ART ranges from 37% to 59% [3, 6]. Historically, the Thai HIV/AIDS Epidemic was centred in Chiang Mai, and several large hospitals provided continuous care for AIDS patients/people living with HIV until effective antiretroviral drugs became available. The province has become a key site for the development and implementation of HIV interventions and research, with well‐established collaboration between government and community partners and high service volume. Today, there are approximately 24,224 people living with HIV receiving antiretroviral treatment in Chiang Mai, including a substantial number of older adults living with HIV (OALHIV) [10].

Given the relatively large numbers of people ageing with HIV, early detection of NCI would allow time to intervene to slow functional decline with increasing age [11] and promote quality of life. Currently, there is no universal NCI screening implemented in HIV clinics in Asian countries, including Thailand. It is in large part because the tests are time‐consuming, often require professional administrators and are not prioritized in the HIV treatment guidelines [12]. Screening tools such as the Montreal Cognitive Assessment or a full diagnostic approach to assessment (e.g. use of Frascati criteria to diagnose HIV‐associated neurocognitive disorders) [13] are conducted as part of research studies and in specialist clinics [14, 15]. They typically require professionally trained psychologists using validated tools—further limiting their implementation in less‐resourced settings.

Many people with HIV are reticent to raise issues that prolong clinic visits or result in more medications or referral to non‐HIV healthcare providers (HCPs) [16]. NCI screening also adds to the workload of already overstretched HIV HCPs, which may limit implementation [17]. The World Health Organization has proposed task‐shifting as an approach to redistribute workload and improve the health system's performance [18]. There is a precedent for using peers and community health workers to support task shifting, for example, community health workers in rural Thailand to support chronically unwell patients with diabetes, tuberculosis or mental health problems [19−21]. However, community health workers are often reticent to care for patients living with HIV due to stigma and lack of training, highlighting the need for a peer‐based model. From the early years of the HIV epidemic in Thailand, community engagement began with informal networks including the Thai Network of People Living with HIV/AIDS, who provided peer‐based support, caregiving and treatment‐related assistance [22, 23]. They were subsequently linked to formal hospital‐based systems from the 2000s onward and later accredited and registered under the National Health Security Office for direct reimbursement of their services. Thus, HIV clinics and hospitals have encouraged people living with HIV to become peer educators to make home visits, help counsel those with new HIV diagnoses about ART and side effects, and encourage care retention. There was no specific requirement for background characteristics. Many adults living with HIV who attended clinics in Northern Thailand were actively involved. With lived experience and practical guidance, task‐shifting support scales up, improves access and reduces costs for certain healthcare services in low‐ and middle‐income countries, particularly for those living with chronic conditions like HIV, which can enhance the quality of care they receive [24, 25].

We employed a multiple‐methods design to explore facilitators and barriers to implementing a peer‐led NCI screening tool among OALHIV aged ≥50 at an HIV clinic in a community hospital in Thailand. We hypothesized that this approach could facilitate the identification of NCI and linkage to further evaluation, diagnosis, and treatment while having a limited impact on the workload of clinic staff.

## 2 Methods

### 2.1 Study Setting

We conducted a multiple‐methods study from March to June 2023 to evaluate the implementation of a peer‐led screening for NCI in an HIV clinic at a community hospital in suburban Chiang Mai Province. At the time of the study, 53% of patients were over 50, and the clinic had five nurses, two nurse assistants and three psychologists providing routine HIV care and other clinical management following clinical practice guidelines prepared by a multidisciplinary care team. Peer educators helped with pill counts, health education and scheduling, but had no involvement with screening or clinical evaluation. There was no full‐time medical doctor nor psychiatrist at the clinic.

### 2.2 Conceptual Framework

We used the Consolidated Framework for Implementation Research (CFIR) to guide the study design and analysis and to inform interview guide development [26]. We adapted CFIR to focus on five domains relevant to this study setting to explore facilitators and barriers to implementation of the NCI screening. The innovation domain included the peer‐led NCI screening intervention; the outer setting included other hospital departments, upper‐level providers and national policies; the inner settings included the clinic space, time spent and workload; individual characteristics included factors related to HCPs, peer educators and OALHIV; and the implementation process domain included services, consultation and referral.

### 2.3 Quantitative Study Procedures and Measures

**FIGURE 1:**
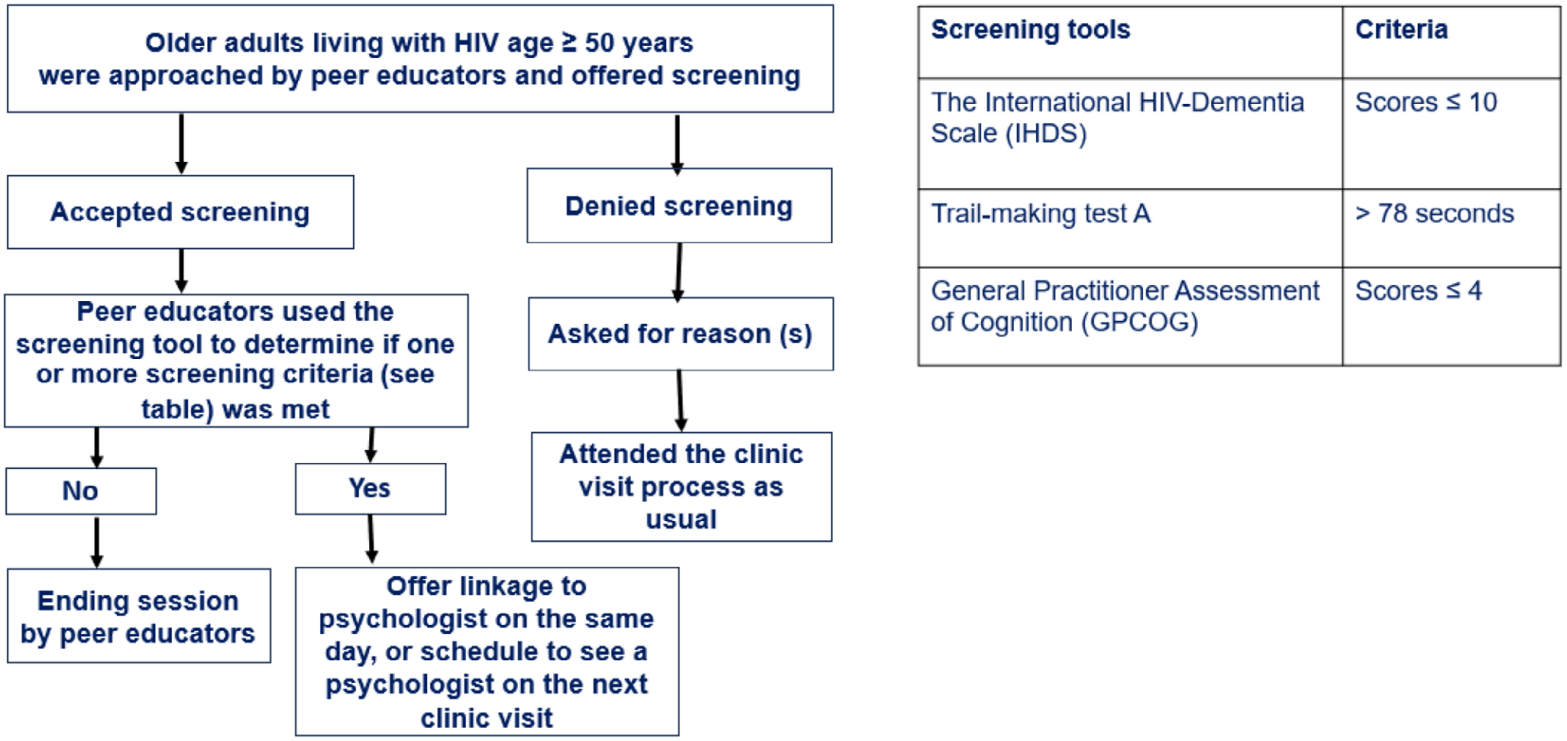
Innovation: Screening process and linkage to psychological evaluation in HIV clinic.

The goal of the first phase was to implement a screening tool for NCI and track its implementation in the clinic. Based on the specific goal of assessing, we used a combination of three easily scored paper‐based tools for use in this study as follows: (1) the International HIV‐Dementia Scale (IHDS) for global cognition [27]; (2) the Trail‐making Test for psychomotor speed; and (3) the General Practitioner Assessment of Cognition (GPCOG). The IHDS and GPCOG have been previously used in Thai populations [28, 29]; the Trail Making Test was selected based on its prior use in Thai HIV cohorts [30], and the feasibility of implementation in a clinical setting as it is paper‐based. Study team members with experience using these measures provided training to HIV peer educators on NCI and how to conduct screening, score results and engage with OALHIV to explain the process. There were five peer educators included, all were female, aged between 50 and 56 years, living with HIV for > 20 years. They practiced using the tool during a 4‐week trial period with supervision from a senior nurse in the clinic. The study was subsequently implemented over a 12‐week period. Trained peer educators approached OALHIV patients aged ≥50 years to participate, and those with positive screening results were linked to HCPs for further assessment (Figure 1).

We conducted descriptive analyses on the numbers of OALHIV who were (1) approached, (2) agreed to complete the screening, (3) had positive NCI screens, (4) accepted linkage for further evaluation by a psychologist and (5) were successfully linked. The validated thresholds for a positive NCI screen for the individual measures used in the screening tool were as follows: IHDS score ≤10 [31], Trail‐making Test A >78 s [32] and GPCOG score ≤4 [29]. The results of evaluations by psychologists and final clinical diagnoses were extracted from medical records.

### 2.4 Qualitative Study Procedures and Measures

We followed the Consolidated criteria for Reporting Qualitative research (COREQ) Checklist to report the study implementation and findings [33]. We conducted interviews with a subsample of OALHIV aged <65 years and 65 years or older who underwent the screening, those who declined screening, peer educators and HCPs to explore the feasibility and acceptability of implementing the screening tool. For OALHIV, we sampled every fifth patient in the screening logbook to ensure consistent representation of participants across the entire screening period and to better mirror the overall screening population. In addition, we also purposefully monitored and filled participant spaces to guarantee representation across age categories, including those 50−65 and over 65 years. If anyone declined, we approached the subsequent patient in the logbook. Provider interviews included all five peer educators who participated in the study. We purposively selected a subsample of six out of 10 HCPs in the clinic to cover a range of professional categories, including psychologists, psychiatric nurses, HIV nurses and the head nurse who supervised the clinic operation. We also interviewed the hospital's head nurse and a primary medical consultant.

All participants were informed about the study and consented prior to the interview. The interview guide was informed by prior research on OALHIV, NCI, and the CFIR domains and related constructs (see Table S2 for examples of questions for each domain). In‐depth interviews were conducted in private spaces at the HIV clinic to ensure confidentiality; each participant was interviewed once. The interviews were conducted in the Northern Thai language by the lead investigator and three research nurses with experience in qualitative interviews, none of whom were HCPs at the clinic. Interviewers wrote field notes after each interview.

**FIGURE 2:**
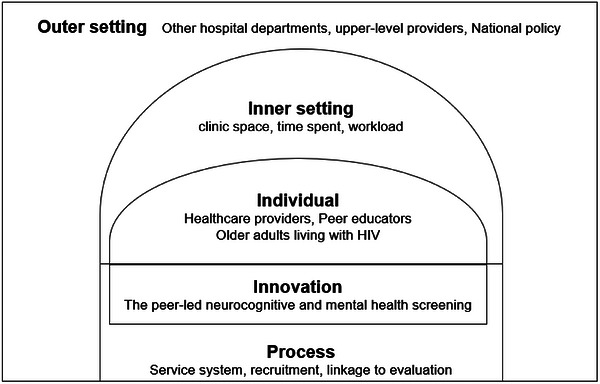
The Consolidated Framework for Implementation Research (CFIR) domain definition for this study.

The digital files were transcribed and reviewed for accuracy by the interviewers. The files were then imported into Dedoose (version 9.2.12). We applied a thematic analysis approach, including line‐by‐line coding [34]. Two interviewers (LA and RT) reviewed all transcripts to become familiar with the data. We then developed a codebook based on the CFIR framework (Figure 2), existing research on this topic, the study interview guide and emergent themes from the transcripts. This codebook was iteratively tested on the first five transcripts and developed and further adapted as new themes emerged. The codebook was presented to the study team during bimonthly meetings to discuss and refine the codes; data saturation was discussed in weekly study team meetings. Once the codebook was finalized, two team members used it to double‐code the remaining transcripts. Coded excerpts were categorized into themes based on relevant CFIR domain constructs and facilitators or barriers to implementation of the intervention. All analyses were conducted in Thai, with selected quotes translated into English to support data presentation.

## 3 Results

From March to June 2023, peer educators approached 162 OALHIV who met eligibility criteria; 144 (89%) agreed to be screened (Table 1). Their median age was 58 years (IQR 54, 62), and 93 (65%) were female. All could verbally communicate in Thai. Among those who were screened, 40 (28%) met the study‐defined cut‐points for referral on at least one of the measures; 31/40 (78%) were linked for further assessment by a clinic‐based psychologist on the same day, and the other nine people were further evaluated during their subsequent visits due to time constraints on the initial screening day. The psychologist reassessed 18 people using the Thai Mental State Examination and 12 people using the Montreal Cognitive Assessment. A diagnosis of mild NCI was made in 20, while another 10 were assessed as within the normal threshold. The overall frequency of asymptomatic NCI was consequently 14% (20 of 144).

**TABLE 1:** Characteristics of older adults living with HIV who were approached and underwent neurocognitive screening.

	Approached for screening	Accepted screening			
Characteristics	Total	Total	Met the screening criteria	Did not meet the screening criteria	Denied screening
Number	162	144 (89%)	44 (27%)	100 (62%)	18 (11%)
Female sex (%)	96 (59%)	93 (65%)	31 (70%)	62 (62%)	6 (3%)
Median age (IQR), years	58 (50−73)	58 (54−62)	61 (51−73)	57 (50−71)	57 (50−70)
Qualitative interviewed participants	26	20 (14%)	11 (25%)	9 (9%)	6 (33%)

We conducted 39 interviews, including 26 with OALHIV and 13 with clinic‐based staff (five peer educators and eight HCPs). The interviews ranged from 17 to 40 min for OALHIV and 33 to 70 min for clinic‐based staff (see Table S1 for their demographic characteristics). Findings are organized to describe facilitators and barriers to implementation (Table 2).

**TABLE 2:** Qualitative themes associated with CFIR domains and constructs.

Domains	Constructs	Facilitators/barriers	Themes
Innovation	Complexity	Barrier	Concern on the screening quality
	Local attitude	Facilitator	Foreseeing benefits of neurocognitive screening
Outer setting	Performance measurement pressure	Barrier	No supportive national policy
Inner setting	Availability	Facilitator	Appreciation of the confidentiality and privacy in the clinic
	Relative priority	Barrier	Clinic‐based time challenges
		Barrier	Low service priority
Individual	Team members	Facilitator	Foreseeing benefits of neurocognitive screening
	Deliverers/motivation	Facilitator	Building on existing relationship
	Deliverers/motivation	Facilitator	Enthusiasm of peer educators to take a screening role
	Recipients	Barrier	Low service priority
	Recipients/need	Barrier	Lack of perceived need
Process	Engaging	Facilitator	Appreciation of the screening and linkage within the clinic with privacy

## 4 Facilitators of Peer‐Led Screening for NCI

### 4.1 Benefits of Neurocognitive Screening

Clinic nurses identified the benefits of, and need for, the screening tools. Knowing about patients’ functional impairment enables them to focus more on counselling and support. They also reported an increase in people with memory problems, particularly as the patient population aged.

*“Lately I have seen more HIV patients in their fifties with memory problems.”* [HCP103, HIV nurse, 36 years]

In addition to patients already identified as having memory problems, participation in this study allowed nurses to see how some OALHIV who have never complained about their memory function met the screening criteria for NCI.

The providers highlighted numerous reasons why this type of screening is beneficial for OALHIV. First, they agreed that HIV or HIV‐related stress might affect brain function:

*“I think people with HIV might have poorer concentration due to stress. I mean, they were anxious about their own health. It could affect their brain.”* [HCP108, HIV nurse, female, 45 years]

Second, a provider mentioned how universal screening could identify patients who might otherwise be missed until the symptoms were quite severe, and it was too late to intervene. She stated that detecting patients as early as possible would maximize the possibility of intervention and slow NCI‐related functional decline.

*“If we can detect cases with NCI early, brief advice like snapshot counseling could be offered by trained providers at the outpatient department. Besides drug prescriptions, occupational therapists or psychologists could help in advising their relatives or caregivers on how to manage at home.”* [HCP107, primary medical consultant, female, 47 years]

### 4.2 Peer‐Educator Empowerment Through Screening Implementation

Peer educators felt confident that they could implement the screening after the training and were enthusiastic to learn how to approach people and conduct the screening session. Involvement in the screening process gave peer educators a feeling of fulfilment since it allowed them to complete more complicated tasks in the clinic. One described receiving positive feedback after she offered the screening.

*“Most patients were happy with me. I could see their faces during the screening. If they did not understand what to do, I would explain a little bit more, and they were OK. No one seemed to be anxious about it.”* [PE101, female, 53 years]

A peer stated that she was unsure about how to approach, conduct and end the screening session the first few times she used the tool, but over time, she gained more confidence after working together with the nurse who supervised them.

*“At first, I was confused about the test. I assigned zero for those who could not recall words by themselves, without providing any clues. During the conversation with the senior nurse who supervised us, I relearned and could do it correctly thereafter.”* [PE111, female, 54 years]

Peer educators mentioned the discussion about the assigned task in their group meeting. They exchanged and learned from one another, which helped them to improve their performance.

*“We talked about the screening in our group meeting. For myself, I consulted our group leader about the screening when I conducted my first few cases. She advised me, and I better understood. None of us complain about the burden of screening in the clinic.”* [PE106, female, 50 years]

While there were some initial challenges, peer educators felt like they improved after working with the supervising nurse. Peer educators discussed issues about their assigned task in their group meeting. They shared experiences and learned from one another, which helped them improve their performance.

*“At first it was difficult to me… I did not know how to say it; I mean to ask questions with big words. However, after several cases, I have more confidence. Discussion with the senior nurse and among our group also helps me a lot.” [PE104, female, 55 years]*

### 4.3 Building on Existing Relationships

Peer educators mentioned that a high percentage of OALHIV agreed to complete the screening, likely because of their familiarity with patients: most OALHIV lived in villages around the hospital area and knew the peers from the clinic and communities.

*“I am familiar with that lady, as I used to be a part of the peer support group in the past. Now many of us quit activities and led our own lives.”* [OA085, female, 56 years]

This sentiment was seconded by OALHIV, who shared how important it is to have a peer‐led programme staffed by people they know.

*“I said YES because it was a kind of…hospital program set up for us. In fact, I knew the one who introduced it to me. We knew each other since we were young, and many of us have already passed away.” [OA147, male, 62 years]*

However, the peer educators’ tasks were not that simple, and some patients could make them feel uncomfortable.

*“I am familiar with most patients in the clinic. Some have close relationships with us, but some only know who we are. Those who did not know us well seemed to be uneasy, like they were uncomfortable while sitting with us doing the test.”* [PE106, female 50 years]

### 4.4 Screening Acceptability Among Patients

OALHIV expressed satisfaction with the screening offered in the clinic and highlighted their appreciation of its privacy.

*“We sit over there, only two of us at the table. It was private without interruption.” [OA147, male 62 years]*

Patients also shared how they spent many hours in the clinic and that they appreciated having the peer‐led NCI screening to fill the gap. This also potentially increased participation rates since OALHIV felt like it was worth trying while they were waiting for HIV care.

*“The peer group leader invited me, and I agreed immediately. I thought it was better than just sitting there. I also am keen to learn what they were doing.” [OA059, male, 70 years]*

Overall, OALHIV described the peer‐led screening as feasible and acceptable. OALHIV who met the NCI cut‐off scores and were linked to care described the linkage process as straightforward, particularly since the HIV and mental health clinics are co‐located, and both teams allowed walk‐ins. An OALHIV described her experience and how she ended up with treatment and felt positive about it.

*“The screening was not too long for me, but I did it badly, and she told me that I needed to see a psychologist. She sent me there, and I was tested again. The psychologist booked a consultant for me for the following month. I came again and got medication, like vitamins for my brain. It made me sleep well.” [OA049, female, 72 years]*

## 5 Barriers

The participants described several barriers to the implementation of the screening tools, many of which aligned with the quantitative results. Lack of time (*n* = 15; 83%) and other medical concerns (*n* = 2; 11%) were the reasons given for refusing screening; another one refused without a specific reason (*n* = 1; 5.5%). No one mentioned specific concerns about the purpose of the screening.

### 5.1 Low Priority and Perceived Need

Many people said they would have done the screening if they had been told in advance of their appointment, but time was the main reason for refusal.

*“She asked if I want to do the screening test. I said I could not. My nephew would pick me up. She should have told me in advance so that I could have organized my schedule accordingly.”* [OA095, male, 59 years, denied]

A second reason was that some OALHIV felt good about their health and did not believe they had any impairment.

*“I work in the garden all day. It is like I already exercise regularly, and I have no memory problem. I always write down every important item to do.”* [OA026, male, 61 years, denied]

Most OALHIV believed that NCI was associated with increased age, not HIV or patterns of ART use. They claimed that their friends without HIV also faced similar problems with memory function. They were not worried, as they saw age‐related memory problems as natural.

*“I don't think about any effects of the drugs, or the virus. I have some memory problems, and my friends without HIV have them too.” [OA127, female, 56 years]*

This belief that any memory issues were normal meant that some people did not accept the screening since they did not see it as necessary.

### 5.2 Concerns Around Screening Quality

Given that 23 of the 44 participants (52%) who met screening criteria for NCI did not meet criteria for impairment when further evaluated by psychologists, the clinic‐based HCPs expressed concern about the quality of the screening and the training of the peers conducting it. Specifically, psychologists expressed their uncertainty about the validity and accuracy of the screening results when done by peer educators.

*“Screening was good, as we would get some more information to advise each patient. However, I was worried about the validity. I think peers need more training, including basic knowledge on dementia.”* [HCP102, psychologist, female, 28 years]

Some HCPs mentioned that alternative approaches to peers conducting the screenings, specifically nurse assistants who had more formal education, were hired by the hospital, and were a part of the healthcare team and thus sufficiently trained.

*“I would advise having our nurse assistants do the screening instead of a peer educator. They have been working closely with healthcare professionals and attended all meetings and conferences with us. They were more educated, and it would be easier for us to provide feedback for improvement, as they were a part of our healthcare team, while peers were volunteers.”* [HCP102, female, 28 years]

Peer educators agreed that more training was needed and that a half‐day workshop was not sufficient to learn and digest things before starting the assigned task.

*“The training session was too brief. I wish they would ask us who wants to do it and who does not. We are not alike. They should train and supervise each of us until we are ready to do it by ourselves. We need more time to practice before we start. They might have to assess, I mean to test if we were capable of doing it, before letting us go to screen others.”* [PE104, female, 56 years]

Both HCPs and peer educators thought peer educators needed additional practice and assessments to ensure they were ready to implement the screening tool.

### 5.3 Challenges Due to the Length of the Screening Tools

There are many activities that HCPs and peer educators are responsible for within the clinic, and many steps for each patient to complete during the visit. Their workload generated worries about screening duration, particularly for HCPs who serve a high volume of patients. They described wanting everyone to complete the clinical services needed and go home as fast as possible.

*“At present, the ratio of providers and clients is imbalanced. I have been working here for 5 years and 4 months. When I started, we had one psychiatrist, then we had two, but now we have none. They all moved away. Our tasks and responsibilities increased; we need to cover both urgent and non‐urgent cases.”* [HCP102, psychologist female, 28 years]

Given the high volume of work in the clinic, HCPs expressed a need for a single, simplified screening tool with fewer questions. They wanted a screener that could be completed in a few minutes, which would be more user‐friendly.

*“I think it is excellent, but it is quite lengthy. We need a tool that can be done in a shorter period.”* [HCP103, HIV nurse]

### 5.4 Low Service Priority in the Absence of Supportive National Policies

HCPs agreed that screening for NCI symptoms in OALHIV is a beneficial initiative. However, it was challenging for them to prioritize since it is not mandated at a national level and thus something they could list as an accomplishment and “key performance indicator” in an annual report.

*“The clinic staff can screen every client if they are assigned to it. Now they have a ton of other assignments and tasks to complete daily.”* [HCP107, Community psychological physician, female 47 years]

As a provider noted, while implementation is possible, it is less likely to be feasible and sustainable until it is mandated.

*“To implement the NCI screening in patients with chronic disease before 60 years of age, we need a national‐level policy. I mean, like a program from the Ministry of Public Health.”* [HCP109, psychological nurse, female, 37 years]

Some providers highlighted that this type of study was important to demonstrate the need for NCI screening. This data could then be used to argue for implementing this type of screening into the national health programme for HIV care, thus making it a priority.

## 6 Discussion

In this pilot study, we demonstrated that a peer‐led NCI screening could be implemented and was acceptable to participants in a busy HIV clinic. We coupled the screening with in‐depth interviews to examine relevant benefits and facilitators to successful implementation of peer‐led NCI screening for OALHIV in a clinic in northern Thailand. For example, HCPs identified key benefits of the screening (e.g. early detection and increased opportunity for diagnosis); peer educators showed enthusiasm for expanding their role in the clinic and identified strong existing relationships between peer educators and patients as a facilitator of screening. All promoted uptake of the screening by patients, peers and HCPs. However, addressing these challenges is necessary to increase the feasibility of task‐shifting.

While we could identify a few studies in Southeast Asia that described task‐shifting NCI screening to non‐HCPs, evidence from mental health and psychosocial support intervention studies in high‐ and low‐middle‐income settings suggests this approach is feasible [35]. Task‐shifting peer educators to screen saved time. The previous two decades have shown that peer educators can help HIV prevention, treatment and retention [36]. When we introduced the screening, all clinics’ peer educators were supportive of being part of the intervention. They appreciated the opportunity to increase their clinical responsibilities and felt a greater sense of agency by having a role traditionally limited to professional HCPs. However, some peer educators expressed anxiety while performing the screening. We perceived their need for our support and approval for them to continue. Another challenge was the internalized HIV‐related and ageing‐related stigma, which might result in incomplete disclosure by OALHIV about their cognitive ability. Besides their supportive manner, the peers have received some practical guidance during the training on how to approach OALHIV like a professional to create trust, including techniques for effective communication and understanding the unique challenges faced by individuals living with HIV.

OALHIV were unaware of the increasing NCI risk associated with ageing, despite high screening uptake. The result was slightly different from a study in the UK where people living with HIV (PLHIV) aged 30−79 years expressed their concern about cognitive issues; however, a similar part was the concern of HCPs regarding the capacity of HIV services to implement routine screening for cognitive impairment [17]. The low NCI awareness in our study might be due to lifestyle and Asian culture that older people get a lot of support from other family members [37]. HCPs knew NCI screening was important, but other clinic services took precedence. HCPs mentioned the time constraint in the clinic, which was in line with a previous Spanish study [38]. They reported that HCPs were aware of neuropsychiatric comorbidities in PLHIV, but there were several reasons for underdiagnosis, including a lack of proactive attitudes, insufficient special training and limited visit time. They emphasized the need for a screening tool that was shorter and easier to administer in the clinic. However, most studies using brief questionnaires for NCI screening among elderly patients had poor sensitivity and specificity compared to a full battery of neurocognitive instruments [39, 40]. HCPs suggested adding pre‐screening questions to the clinic intake form for all OALHIV. Positive answers could indicate further NCI screening, which would save time for everyone. In the current study, HCPs worried about screening delivery and score interpretation, as we did not offer full training to them. False‐positive NCI results were a source of frustration for several HCPs in this study, as they were asked to confirm the presence of NCI; other research suggests that HCPs may be unfamiliar with the emphasis on high sensitivity in screening approaches designed to minimize missed cases of NCI [41]. It is important that all relevant staff be made aware of the implementation.

Our study results presented several learning experiences that might inform future NCI screening in similar settings. First, we should provide options for peer educators to choose whether or not to participate. The second is the need for comprehensive training for non‐provider screeners, including knowledge and ability checks before assigning tasks and continual quality control. Training could be separated into several sessions with pre‐ and post‐evaluation. Third, we should include HCPs in the planning phase before implementation to enhance their sense of validity regarding the screening process. Screening assessments could be tailored to clinic providers’ preferences, allowing for either brief questions or paper‐based tools, based on agreements with HCPs.

Currently, there is no routine NCI screening for OALHIV, while Thailand's national health policy limits neurocognitive screening to people 60 years or older [42], regardless of HIV serostatus. Given the rising evidence of earlier NCI development in people living with HIV, a Chinese study screened NCI in OALHIV aged 50 years and older and reported a significantly higher prevalence of NCI when compared to the non‐HIV population [43]. OALHIV screening should begin earlier in HIV than in the general population. Awareness of NCI among HCPs, patients and caregivers is necessary for timely assessment, diagnosis, and intervention, including pharmaceutical and non‐pharmacological treatments. Increasing patient education on ageing‐related health issues and sharing study findings with HCPs would help them recognize the relevance of assessing and controlling NCI in OALHIV. Our findings support the feasibility and acceptability of NCI screening in HIV clinics. Revised clinical guidelines that endorse NCI screening for OALHIV may help shift how healthcare policymakers think about NCI and HIV, leading them to modify Thailand's national AIDS programme to cover routine NCI screening, which could ultimately improve health outcomes for OALHIV.

There are several study limitations. First, the relatively short implementation period prevented us from examining the potential long‐term benefits of routine NCI screening on clinical outcomes, as well as assessing the sustainability of integrating screening over time. Second, the peer educators received only a brief training in the use of the screening tool. No formal assessment of screening skills was done, and variations in the use of the tool could have affected the results. Third, the study was conducted in a well‐resourced rural healthcare facility, and results may not apply to clinics in different contexts. Future research on NCI screening implementation might consider brief screening questions to be asked during vital sign measurement in the clinic, or alternatively, artificial intelligence might be applied for a rapid NCI screening. A similar implementation of peer‐led NCI screening, like we did, could be further researched for feasibility and acceptability in other clinics with different settings, like those with younger peer educators or clients with higher education in urban areas.

## 7 Conclusions

Our study demonstrated that a peer‐led NCI screening intervention was feasible and acceptable in a hospital‐based HIV clinic. Key facilitators included the strength of existing relationships between peer educators and OALHIV and their trustworthiness in the health services provided. HCPs acknowledged the advantages of peer‐led screening; however, they also recognized obstacles, such as time constraints and concerns regarding screening quality, which could impede the overall efficacy of the intervention and the perceived benefit of the NCI implementation. Engagement with HCPs and patients across all stages, from planning to implementation and evaluation, as well as more intensive training and mentorship, could overcome some of the identified barriers. Expanding screening for OALHIV by integrating into the routine HIV care aims to identify cases of NCI as early as possible, thereby facilitating care engagement that leads to long‐term quality of life.

## Author Contributions

LA and NP conceptualized the study aims and design. PT, MMP, and CAM reviewed and provided input into the aims and design. LA, RT and AT conducted the study, collected quantitative and qualitative data. PT and RT recruited study participants. LA, RT, and AT analysed and interpreted data. NP, MMP and CAM made critical revisions to the manuscript. All reviewed and approved the final version.

## Funding

This work was supported through a grant from amfAR, The Foundation for AIDS Research, with support from the US National Institutes of Health's Fogarty International Center and the National Institute of Mental Health (CHIMERA; D43TW011302).

## Conflicts of Interest

The authors have no conflicts of interest.

## Disclaimer

This work is solely the responsibility of the authors and does not necessarily represent the official views of any of the institutions mentioned above.

## Ethics Statement

The study was approved by the Human Experimentation Committee, Office of Research Ethics of the Research Institute for Health Sciences, Chiang Mai University (certificate of approval no. 02/2023). The study was conducted in accordance with the local requirements. The study did not consider OALHIV in the clinic who underwent the peer‐led screening as study participants. All interviewed participants provided written informed consent, and we provided USD 16 to IDI participants to reimburse them for their time.

## Supporting information




**Table S1**: Demographic characteristics of in‐depth interview participants
**Table S2**: Example of interview questions based on Consolidated Framework for Implementation Research

## Data Availability

The data that support the findings of this study are available on request from the corresponding author.
