# Supplementary material for: Facilitators and Barriers to Implementing Peer‐Led Neurocognitive Screening for Older Adults Living With HIV at a Community Hospital in Thailand: A Multiple‐Methods Study
Source: J Int AIDS Soc. 2026 Jun 11;29(6):e70135. doi: 10.1002/jia2.70135 (PMC13260682; doi:10.1002/jia2.70135)
Supplement: Supplementary file 1 — Table S1: Demographic characteristics of in‐depth interview participants Table S2: Example of interview questions based on Consolidated Framework for Implementation Research [file JIA2-29-e70135-s001.docx]

| **Supplementary table 1 Demographic characteristics of in-depth interview participants** | | | |
| --- | --- | --- | --- |
| **Characteristics** | **OALHIV** | **Healthcare providers ^a^** | **Peer educators ^b^** |
|  | **n=26** | **n=8** | **n=5** |
| Female sex | 15 (58%) | 8 (100%) |  |
| Median age (range), years | 62 (53-73) | 41 (28-58) | 54 (50-56) |
| Highest education |  |  |  |
| Primary school | 25 (96%) | 0 |  |
| Secondary school | 1 (4%) | 0 |  |
| Bachelor's degree or higher level | 0 | 8 (100%) |  |
| Duration in the clinic ^c^ |  |  |  |
| < 2 years | 0 | 1 (13%) |  |
| 2-4 years | 2 (7.7%) | 1 (13%) |  |
| 5-9 years | 1 (3.8%) | 3 (38%) |  |
| 10-19 years | 17 (65%) | 1 (13%) |  |
| 20 years or more | 6 (23%) | 2 (25%) | 5 (100%) |
| *^a^ Two HIV nurses, two psychiatric nurses, two psychologists, two hospital staff in leadership roles).*  *^b^ Sex and education are not shown due to small number of participants in this category.*  *^c^ For antiretroviral treatment or working in the current position* | | | |
| *OALHIV older adults living with HIV* | | | |

**Supplementary table 2 Example of interview questions based on Consolidated Framework for Implementation Research**

| **CFIR domains** | **Informants** | **Example of questions** |
| --- | --- | --- |
| **Intervention** | OALHIV-screened | -What do you think about the screening? (questions, tasks, duration, like or dislike) |
|  | OALHIV-declined | -What was your main reason for declining screening?  -Can you describe your previous experience being screened, if any? |
|  | Healthcare providers | -The like/dislike about the screening  -How to improve acceptability? |
|  | Peer educators | -How was it going on?  -What is your impression about the screening form?  -What do you think about the screening? (questions, tasks) |
| **Outer setting** | OALHIV-screened | -Have you shared your screening experience with your friends or family members? What were their reactions?  -Do you think anyone will be willing to pay for the screening, why or why not? |
|  | OALHIV-declined | -Have you heard any bad things about the screening? What are they?  -Have you heard from anyone about the need for NCI screening? Good or bad |
|  | Healthcare providers | -Do you think the national program officer will like this intervention? Why or why not? |
|  | Peer educators | -Do you think the national program officer will like this intervention? Why or why not? |
| **Inner setting** | OALHIV-screened | -Do you think it is appropriate to do the screening in clinic?  -Can you think of any other places that screening could be done? |
|  | OALHIV-declined | Will you be interested in being screened if it occurs at other places, other times, or by other people? Please share your thought. |
|  | Healthcare providers | -Whether the screening should take place in the clinic or anywhere else, where are they?  -Should it be done in all clients? How frequent?  -Interfere with routine clinic activities? (time, staff, space etc.) |
|  | Peer educators | How comfortable were you while offering the screening? What are the barriers?  -what made the task easier or more challenging? |
| **Individuals** | OALHIV-screened | What is your idea about NCI and MH in OALHIV?  -Do you think you have any related problems before the screening? Describe your experiences |
|  | OALHIV-declined | -What is your idea about NCI and MH in OALHIV?  -Do you think you have any problems before the screening? Describe your experiences |
|  | Healthcare providers | -What are your roles in clinic/working with PLHIV?  -Can you provide some thoughts about the national HIV care program?  -Do you think PLHIV have more NCI than general population?  -What do you think about the NCI screening? |
|  | Peer educators | -What are your roles in clinic/working with PLHIV?  -Can you provide some thoughts about the national HIV care program?  -Do you think PLHIV have more NCI than general population?  -What do you think about the NCI screening? |
| **Process** | OALHIV-screened | -What do you think about their approach when asking you to join the screening?  -How did they advise you after the results were out? How do you feel about it? |
|  | OALHIV-declined | Not applicable |
|  | Healthcare providers | -Can you provide comments about the process (approach, procedure, duration, and anything about the screening), from your own thought or what you have heard from others? |
|  | Peer educators | -How difficult was it to ask OALHIV to join the screening?  -What do you think about the duration to complete one?  -How did your clients react during the test? Do you think they like it? |

CFIR the Consolidated Framework for Implementation Research; OALHIV older adults living with HIV; PLHIV people living with HIV; NCI neurocognitive impairment; MH mental health
